# 克唑替尼治疗晚期NSCLC患者单中心回顾性分析

**DOI:** 10.3779/j.issn.1009-3419.2016.03.07

**Published:** 2016-03-20

**Authors:** 峨嵋 高, 军 赵, 明磊 卓, 志杰 王, 玉艳 王, 彤同 安, 梅娜 吴, 雪 杨, 佳 仲

**Affiliations:** 100142 北京，北京肿瘤医院胸内一科 Department of Respiratory Oncology, Beijing Cancer Hospital, Beijing 100142, China

**Keywords:** 肺肿瘤, 间变淋巴瘤激酶, 克唑替尼, Lung neoplasms, Anaplastic lymphoma kinase, Crizotinib

## Abstract

**背景与目的:**

克唑替尼是针对间变淋巴瘤激酶（anaplastic lymphoma kinase, *ALK*）融合基因、ROS-1重排等靶点的药物。本文观察克唑替尼治疗ALK/ROS1重排阳性的晚期非小细胞肺癌（non-small cell lung cancer, NSCLC）的近远期疗效。

**方法:**

对2013年6月-2014年12月于北京肿瘤医院开始接受克唑替尼治疗的40例相应靶点阳性的NSCLC患者进行回顾性分析。

**结果:**

本组40例，39例为腺癌或腺鳞癌，包括印戒细胞癌、腺泡型、乳头状腺癌等特点。中位年龄49.5岁，治疗总有效率62.5%，疾病控制率为95.0%。全组中位随访14.6个月，中位无进展生存时间（progression free survival, PFS）7.5个月，中位生存期（overall survival, OS）尚未到达，1年生存率77.4%。第一、二线较二线后治疗者中位PFS、OS有延长趋势，但无统计学意义（PFS: 9 mo *vs* 6 mo, *P*=0.06; OS: 21.5 mo *vs* 14.6 mo, *P*=0.12）。20例进展者以脑转移为进展部位。进展后接受二代/三代ALK-酪氨酸激酶抑制剂（tyrosine kinase inhibitor, TKI）的患者表现出疾病控制、生存延长的疗效。不良反应主要为消化道反应、转氨酶升高、特征性的视觉异常等。

**结论:**

本组克唑替尼治疗相应靶点阳性晚期NSCLC临床特点、疗效及不良反应与国际报道相近。脑转移进展是克唑替尼治疗后进展的常见形式，克唑替尼耐药者给予二代/三代ALK-TKI可延迟进展。

## Abstract (en)

**Background and objective:**

Crizotinib was developed in recent years based on targets of anaplastic lymphoma kinase (*ALK*) fusion genes. The aim of this study is to explore the efficacy of crizotinib in treatment of non-small cell lung cancer (NSCLC) with ALK/ROS1 rearrangement.

**Methods:**

Retrospective analysis of 40 patients with ALK/ROS1-positive NSCLC, who received treatment in Beijing Cancer Hospital during the period from Jun. 2013 to Dec. 2014.

**Results:**

Among these cases, 39 were adenocarcinoma and adenosquamous carcinoma, with characters involving signet-ring cell carcinoma, polypoid adenocarcinoma, acini and papillary adenocarcinoma. The median age was 49.5 years old, with the overall response rate of 62.5% and disease control rate of 95.0%. Of all the cases, median follow-up was 14.6 months and median PFS 7.5 months; median OS has not been reached; the one-year survival rate was 77.4%. The median PFS and OS of patients receiving first and second-line treatment tend to be longer than those who received post-second line treatment, but with no statistical significance (PFS: 9 mo *vs* 6 mo, *P*=0.06; OS: 21.5 mo *vs* 14.6 mo, *P*=0.12). Twenty patients who experienced progression in brain metastases. After experiencing progression, the patients receiving 2^nd^/3^rd^ generation ALK-TKI treatment showed efficacy of disease control and survival. The adverse events include gastrointestinal reaction, transaminase elevation, and distinctive visual abnormalities, *etc*.

**Conclusion:**

The clinical features, efficacy, and adverse events of crizotinib in the treatment of the 40 patients with ALK/ROS1-positive NSCLC are similar to the data from the previous reports. The most common site of progression was brain metastases. The treatment of crizotinib-resistant patients using 2^nd^/3^rd^ generation ALK-TKI could delay progression.

肺癌分子靶向治疗的时代开始于2004年对非小细胞肺癌（non-small cell lung cancer, NSCLC）表皮生长因子受体（epidermal growth factor receptor, EGFR）激活突变与EGFR酪氨酸激酶抑制剂（tyrosine kinase inhibitor, TKI）疗效相关的发现。继之，寻找NSCLC其他潜在的驱动突变并研制相应的靶点药物成为研究热点。2007年，两个独立研究组（Soda等^[1]^和Rikova等^[2]^）同时发现了NSCLC中的另一驱动基因——棘皮动物微管相关蛋白4与间变淋巴瘤激酶（echinoderm microtubule associated protein like 4-anaplasticlymphomakinase, *EML4*-*ALK*）基因重排易位融合。这一融合基因作为一种组成性激活形式，驱动细胞内涉及受体酪氨酸激酶的三条主要信号通路（RAS/MEK/ERK, PI3K/AKT, JAK2/STAT3）活化，促进NSCLC的病理形成^[3]^。随后，ALK阳性NSCLC分子亚型被确立^[4]^，通过计算机精确模拟化学结构而研发的针对ALK/MET/ROS-1靶点的小分子TKI药物克唑替尼通过了临床前研究及临床逐期试验，证实其明显优于化疗的疗效及安全性^[3]^。

基于上述一系列研究的重大成果，2011年8月美国食品与药物管理局批准克唑替尼上市^[3, 5-7]^用于治疗ALK重排阳性的NSCLC，这是继EGFR-TKI之后NSCLC靶向治疗的第二个里程碑式的成就^[3]^。2013年，克唑替尼在中国获批上市，同时，中国专家共识将规范化流程的Ventana免疫组织化学（immunohistochemistry, IHC）与用逆转录-聚合酶链反应（reverse transcription-polymerase chain reaction, RT-PCR）共同列为ALK阳性NSCLC诊断方法，开启了我国诊断和治疗ALK阳性NSCLC的新局面^[6]^。经过近2年临床经验的积累，我们对该类疾病的临床特征与治疗方法的理解日渐深入。本研究即对北京肿瘤医院胸内一科收治的40例口服克唑替尼治疗的ALK/ROS-1阳性晚期或局部晚期NSCLC患者临床特征、近远期疗效及安全性进行回顾性分析，以充实我国应用克唑替尼治疗NSCLC实践的临床研究数据。

## 1 资料与方法

### 1.1 纳入标准

组织病理学或细胞学证据的Ⅲb期-Ⅳ期NSCLC（国际肺癌研究协会第7版NSCLC分期标准为据），经Ventana IHC或荧光原位杂交技术（fluorescence in *situ* hybridization, FISH）或RT-PCR基因检测*ALK*重排融合基因阳性（或*ROS1*基因重排检测阳性），接受过至少28天克唑替尼治疗（初始标准剂量250 mg *bid*）；具有客观疗效评估依据，根据实体瘤疗效评价标准（Response Evaluation Criteria in Solid Tumors, RECIST）1.1版进行疗效评估，临床资料完整，纳入生存分析者无门诊或电话失访。

### 1.2 临床资料

本回顾性研究纳入2013年6月-2014年12月间于北京肿瘤医院胸部肿瘤内一科接受克唑替尼治疗的晚期或局部晚期NSCLC患者40例，其中39例经检测*ALK*重排融合基因阳性，2例*ROS*-1基因重排检测阳性（其中1例与*ALK*融合基因阳性共存）。

### 1.3 疗效及不良反应（安全性）评价

所有患者收集治疗前1个月内影像学资料作为基线，克唑替尼治疗1个月后进行首次影像学复查[计算机断层扫描（computed tomography, CT）、磁共振成像（magnetic resonance imaging, MRI）等]，根据RECICT 1.1标准进行疗效评价，之后每2个月定期进行影像学复查及随访。有效率（response rate, RR）定义为客观缓解率，即完全缓解（complete response, CR）加部分缓解（partial response, PR）患者占全部患者的比率。疾病控制率（disease control rate, DCR）定义为CR+PR+SD（肿瘤稳定）患者占全部患者的比率。无进展生存期（progression free survival, PFS）定义为自克唑替尼（或ALK-TKI）治疗开始至出现有客观证据证明疾病进展（或虽未观察到进展但以任何原因死亡）的时间。总生存期（overall survival, OS）定义为自克唑替尼（或ALK-TKI）治疗开始至任何原因死亡的时间。1年生存率定义为自克唑替尼治疗开始计算生存时间满1年的患者比率。数据截止时仍存活的患者以末次随访或末次病历记录的日期作为截止日期。随访截止时间为2015年11月30日。不良反应（安全性）按常见不良反应事件评价标准（CTCAE）4.0版标准进行评价。

### 1.4 统计学方法

应用SAS V8统计软件进行数据处理，近期疗效差异比较应用*Fisher*精确检验，生存分析应用*Kaplan*-*Meier*法作图和*Log*-*rank*检验，以*P*＜0.05为差异具有统计学意义。

## 2 结果

### 2.1 一般资料

本组共40例患者临床及病理特征（表 1）总结如下：男性19例，女性21例，中位年龄49.5岁。不吸烟者占80%，其中Ⅳ期为主（39/40），病理诊断以腺癌为主（39/40）。35例Ventana IHC检测ALK阳性，4例FISH检测ALK阳性，3例RT-PCR检测ALK阳性；2例ROS-1检测阳性（其中1例与ALK共阳性）；2例与*EGFR*突变共存，1例与*KRAS*突变共存。全组患者治疗前胸膜转移者多见（23例），脑转移者11例。全组治疗后出现脑及脑膜转移进展多见（21例）。克唑替尼治疗时机以一二线为主各占13例，三线以上14例。

**1:** 40例ALK/ROS-1重排阳性患者临床及病理、基因检测特征 Clinical and pathological, genetic testing features for 40 patients with ALK/ROS-1 rearrangement

Feature	Classification and cases	
Gender	Male	19
	Female	21
Smoking status	Never smokers	32
	Regular smokers	6
	Occasional smokers or unknown	2
ECOG score	0-1	37
	2	3
Stage	Ⅳ	39
	Ⅲb	1
Age (year)	> 50	16
	≤50	24
Characteristics of metastasis	Pleura effusion/ pericardium	28
	Brain and meninges	11
	Bone	15
	Liver	6
	Adrenal gland	4
Treatment line	1^st^	13
	2^nd^	13
	3^rd^	6
	4^th^	5
	5^th^	2
	6^th^	1

### 2.2 疗效、进展后治疗及生存情况

全组影像学评估克唑替尼总有效率为62.5%（25/40），其中一线治疗有效率达76.9%（10/13），二线治疗有效率为69.2%（9/13），三至多线治疗有效率为42.9%（6/14）。全组疾病控制率为95%（38/40），中位缓解时间为1个月。不同性别、年龄（≤50岁与＞50岁）、吸烟状况（吸烟者与不或少吸烟者）、有无脑转移、各线治疗（一线与二线至多线）之间有效率无统计学差异（*Fisher*精确检验*P*＞0.05）。

40例纳入生存分析，中位随访14.6个月，全组中位PFS 7.5个月；全组中位OS尚未到达（低值21.5个月），1年生存率77.4%。不同性别、年龄（≤50岁与＞50岁）、吸烟状况（吸烟者与不或少吸烟者）、有无脑转移之间PFS及OS无统计学差异。全组无进展生存及生存曲线、一与二线对比二线后治疗者无进展生存及生存曲线见图 1-图 2。如图 2所示，第一、二线治疗者较二线后治疗者中位PFS、OS有延长趋势，但差异无统计学意义（PFS：9个月*vs* 6个月，*Log*-*rank*检验：*P*=0.06；OS：21.5个月*vs* 14.6个月，*Log*-*rank*检验：*P*=0.12）。

**1:**
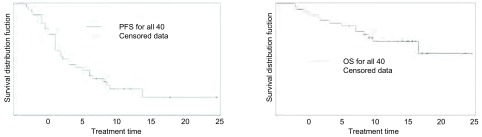
全组40例口服克唑替尼患者PFS曲线（A）和OS曲线（B） PFS (A) and OS (B) curves for all 40 patients receiving crizotinib treatment

**2:**
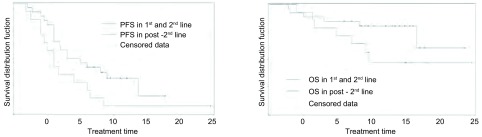
一线、二线对比二线后治疗PFS曲线（A）和OS曲线（B） PFS (A) and OS (B) curves in 1^st^ and 2^nd^ line vs in post-2^nd^ line treatment

至随访截止期全组共有31例患者克唑替尼治疗进展，这些患者的后续治疗包括联合局部治疗（颅脑及肺部病灶放疗、胸水引流及胸腔化疗、肝转移介入治疗等）、继续克唑替尼治疗或更换全身治疗方案（二代/三代ALK-TKI、全身化疗或加用烷化剂化疗等），治疗及转归情况见表 2。以脑转移为主要进展部位者占64.5%（20/31），2例出现脑膜转移。80%（16/20）脑转移进展者在脑部病灶进展时肺部病灶稳定获益，通过给予转移灶或全脑放疗，并继续克唑替尼（耐药者更换新一代ALK-TKI）口服，70%（14/20）仍存活，中位OS尚未到达（低值21.5个月）。

**2:** 31例克唑替尼治疗进展患者进展模式、后续治疗及转归 Clinical modes, subsequent treatments and management outcome for 31 cases in progression diseases with the treatment of crizotinib

Progression modes (cases)	Subsequent treatments classification after crizotinib failure (Cases/Controlled Cases^a^)							mPFS (mo)	mOS(mo)
	Systemic therapy				Local treatment				
	Crizotinib-based	2^nd^/3^rd^ ALK-TKI	Chemotherapy	Symptomatic-based	Local radiotherapy (Brain, lung, bone)	Thoracic cavity treatment	Interventional therapy		
Gradual or local progression (17)	10/8	4/4	4/4^b^	1/ 0	10/9	2/1	0	8.0	Not reached
Dramatic progression or PFS < 3 mo (14)	5/3	5/4^c^	3/1	2/0	5/4	2/0	1/1	5.5	Not reached
a: The controlled cases in each treatment item not only come from a particular therapy but from the combination of systemic therapy and local treatment. b: Of the four patients receiving chemotherapy, two received the treatment of Crizotinib plus alkylating agent and one continued to take Crizotinib after one cycle of systemic chemotherapy, which were all attributed to the comprehensive treatment. c: One uncontrolled patient died only after one-week treatment of 2nd generation ALK-TKI. mPFS: median progression free survival; mOS: median overall survival.									

### 2.3 不良反应（安全性）

本组患者接受克唑替尼治疗后总体安全性较好，常见不良反应（发生率大于10%）为：恶心及呕吐、腹痛及便秘、腹泻、转氨酶升高、视觉异常、中性粒细胞减少、贫血及乏力，多数为1级-2级，3级/4级不良反应包括：恶心及呕吐2例（5%）、腹泻1例（2.5%）、中性粒细胞减少2例（5%）以及转氨酶升高3例（7.5%）。其他少见毒性包括：窦性心动过缓、肢体水肿、神经性麻木、听觉异常（耳鸣、听力下降）、湿疹及皮疹、胆红素升高、白蛋白下降、下肢静脉血栓等。8例患者因消化道反应、窦性心动过缓、粒细胞减少、肝功能异常、血栓形成暂停克唑替尼治疗5天至1个月，2例患者因肝功异常及粒细胞缺乏减量至原剂量50%-80%，无终止治疗患者。汇总克唑替尼不良反应见表 3。

**3:** 应用克唑替尼后与任何原因相关的不良反应（*n*=40） Adverse reactions associated with any reason after crizotinib treatment (*n*=40)

Adverse reactions	Any grade [*n* (%)]	3/4 grade [*n* (%)]	Dose reduction/ temporary withdrawal	Treatment termination
Digestive reaction				
Nausea & vomiting	25 (62.5)	2 (5)	3	0
Abdominal pain and constipation	7 (17.5)	0	0	0
Diarrhea	14 (35)	1 (2.5)	1	0
Disordered vision	17 (42.5)	0	0	0
Neutropenia	7 (17.5)	2 (5)	2	0
Anemia	4 (10)	0	0	0
Transaminase elevation	21 (52.5)	3 (7.5)	2	0
Increased bilirubin	1 (2.5)	0	0	0
Albumin decline	1 (2.5)	0	0	0
Fatigue	4 (10)	0	0	0
Sinus bradycardia	3 (7.5)	0	1	0
Limb edema	3 (7.5)	0	0	0
Nerves numb	2 (5)	0	0	0
Tinnitus, hearing loss	1 (3)	0	0	0
Limb vein thrombosis	3 (7.5)	0	1	0
Rash	3 (7.5)	0	0	0
Lower creatinine clearance	1 (2.5)	0	0	0

## 3 讨论

EML4-ALK融合的发现与治疗2011年被评为临床肿瘤界的十大进展之一，他开启了肺癌第二个靶向治疗的时代^[3]^。从2007年肺癌患者ALK靶点发现到2011年*ALK*融合基因抑制剂克唑替尼成功获批上市治疗，ALK-TKI以4年时间走完了从EGF到EGFR再到EGFR-TKI近乎40年的发展历程，成为转化研究的经典范例^[8]^。

*ALK*重排被认为是一个小概率、大意义的分子事件。许多组织库的回顾性研究证实NSCLC中*ALK*重排的发生率为3%-5%（年轻、女性、从不吸烟的腺癌中这一比率可能升高至8.3%^[9]^）。*ALK*重排NSCLC较*EGFR*突变肺癌更倾向于年轻人（前期回顾性研究和几项大宗研究PROFILE 1001、1005、1007的数据^[3-5]^显示ALK阳性患者诊断时中位年龄均接近50岁-51岁），从不吸烟或少吸烟的比例更高，无明显种族差异，男女无性别偏好，多数呈腺癌，*EGFR*、*KRAS*基因多为野生型。ALK阳性腺癌常见的组织学特征包括：肺腺泡癌、乳头状及微乳头状癌、印戒细胞癌或局限印戒细胞成分的实体型和粘液管状腺癌类型。有研究报道其临床表型倾向于出现更多的心包和胸膜受累^[3]^。

本组研究的40例男女例数相当，中位年龄49.5岁，80%为从不吸烟者，几乎均为腺癌，组织学特征涉及乳头状腺癌、粘液腺癌、印戒细胞癌和以腺泡方式生长的腺癌这些ALK阳性肺癌的易见类型，与*EGFR*、*KRAS*突变共存的病例仅为3例和1例，出现胸膜、胸腔受累者超过半数，这些临床及病理特征均与国际研究的结果相似。

PROFILE 1001和PROFILE 1005分别是克唑替尼全球多中心开放单臂Ⅰ期、Ⅱ期临床试验，相继于2006年、2009年启动，入组可评估疗效患者分别为143例、259例，结果显示既往化疗失败的*ALK*重排NSCLC应用克唑替尼客观有效率高达60%（PROFILE 1001历时4年，历年报道有效率53%-61%，更新数据与其后PROFILE 1005有效率一致，均趋向60%），PFS分别为9.7个月、8.1个月，1年生存率分别为75%、67%^[3, 6, 10, 11]^，远远高于单药化疗在总体人群中缓解率不足10%、PFS仅2个月-3个月的疗效，且疗效获得迅速，半数观察者疗效发生于最初随访的8周内。之后，两项Ⅲ期随机对照临床试验PROFILE 1007、PROFILE 1014相继启动，分别研究二线和一线应用克唑替尼对比化疗治疗ALK阳性NSCLC的疗效。2013年Shaw等在《新英格兰杂志》报道了PROFILE 1007研究结果^[12]^，显示二线应用克唑替尼（173例）对比培美曲塞或多西他塞化疗（174例）总有效率为65% *vs* 20%（*P*＜0.001），中位PFS为7.7个月 *vs* 3.0个月（*P*＜0.001），克唑替尼组近1/3患者病灶缓解超过60%，中位PFS是化疗组的2倍多。2014年Solomon等也在《新英格兰杂志》发表了PROFILE 1014的研究结果^[13]^，证实一线克唑替尼（172例）对比顺铂为基础的化疗（171例）疗效更明显：总有效率74% *vs* 45%（*P*＜0.001），中位PFS 10.9个月 *vs* 7.0个月（*P*＜0.001），化疗组疾病进展后可交叉至克唑替尼组，多数进展者仍在做生存随访，中位OS未到达。这一结果为2014年美国国立综合癌症网络（National Comprehensive Cancer Network, NCCN）指南推荐克唑替尼一线治疗晚期ALK阳性NSCLC奠定了基础。此外，Shaw等^[14]^回顾性研究显示在PROFILE 1001中入组克唑替尼的患者较未入组者有生存的明显改善，中位OS达29.6个月，这是目前化疗无法企及的远期疗效。这些研究中的亚裔患者数据更佳，PFS及OS均优于非亚裔。

在上述国际研究背景下，本研究结果显示40例克唑替尼治疗的总有效率62.5%，一线治疗有效率76.9%，二线治疗有效率69.2%，全组中位PFS 7.5个月，中位OS不少于21.5个月，1年生存率77.4%；此疗效水平与国际研究结果相近，一线、二线较二线后治疗者中位PFS、OS有延长趋势，提示尽早地筛查ALK阳性NSCLC给予一线、二线应用克唑替尼可能疗效更优。

目前国际研究^[3]^认为克唑替尼治疗过程中出现脑转移是一个特殊的临床情况。2012年，Ou等^[3]^基于PROFILE 1001和1005的临床经验指出，应用克唑替尼进展后继续获益的患者有近半数为脑转移患者，考虑克唑替尼在脑脊液中的浓度仅为血浆水平的0.26%（0.001, 4 μmol/L），认为脑的孤立进展反映了他是克唑替尼的“避难圣地”，而非真正的克唑替尼耐药；局部放疗同时继续克唑替尼治疗可为具有寡转移病灶的患者提供额外6个月的PFS。2015年Daniel等总结PROFILE 1005和1007的数据提示：克唑替尼应用12周时全身和颅内转移的控制情况相似（56%-65%），但颅内病灶的中位进展时间明显短于全身基线病灶的中位进展时间，指出在克唑替尼治疗过程中先前存在或新发的颅内病灶进展是一种普遍现象，不同于EGFR-TKI的治疗特征，这可能与*ALK*重排NSCLC中枢神经系统（central nervous system, CNS）的自然病程相关，但他强调这通常是获得性（继发）耐药的表现^[15]^。同时基于回顾性分析：评效进展的患者继续克唑替尼治疗比中止该药治疗有更好的生存获益，他认为孤立性脑转移进展不是获得性耐药的常见表现，可采用继续克唑替尼治疗同时加用脑放疗的策略以延长非进展病灶全身控制的时间^[14]^。而新一代ALK/ROS TKI设计增加了CNS的穿透力和比克唑替尼更强的ALK TKI活性，用于初治和耐药的ALK阳性NSCLC脑转移将更加令人期待。目前一些动物实验和临床研究已证实二/三代ALK TKI在治疗中枢神经系统肿瘤中的优势和在克唑替尼耐药患者中的疗效^[16-19]^。

本研究约27%（11/40）患者存在基线脑转移，在克唑替尼治疗进展的患者中，有64.5%（20/31）出现脑部病灶进展，支持克唑替尼治疗中脑转移的高发生率。对于这部分人群，我们大多采用继续以克唑替尼或序贯二代、三代ALK抑制剂为主的全身治疗+脑转移灶局部放疗，多数患者颅内病灶控制，肺部病灶稳定，生存继续获益，20例脑转移进展患者中位OS尚未到达，目前已超过21.5个月，说明单纯脑转移进展不是停用克唑替尼或ALK-TKI指征，加用局部放疗对继续控制肿瘤意义重大。本组1例脑及脑膜转移进展的原发耐药患者和4例脑转移进展后继发耐药者，在更换二代/三代ALK TKI后都有相应的PFS、OS获益和症状改善，支持新一代ALK-TKI有克服克唑替尼耐药的优势。

克唑替尼最初作为MET抑制剂合成，是ALK/MET/ROS-1多靶点TKI，因此除了ALK重排，*C*-*MET*扩增、突变和*ROS*-*1*重排融合也是克唑替尼的重要靶点。ROS-1是人类58个受体酪氨酸激酶之一，与ALK的演进相关，在NSCLC中的发生率为1%-2%。马萨诸塞州总医院Bergethon等^[20]^2012年报道1, 073例NSCLC肿瘤样本中筛选出*ROS*-*1*重排NSCLC 18例（1.7%），东亚腺癌人群发生率1%，临床特征与*ALK*重排NSCLC相似，克唑替尼治疗有效率57%，8周疾病控制率79%；PROFILE 1001报道*ROS*-*1*重排NSCLC 50例，克唑替尼客观有效率72%，中位PFS 19.2个月，1年生存率85%^[21]^，提示ROS-1重排是ALK-TKI的有效靶点。近来研究^[22, 23]^发现，第三代ALK/ROS-1 TKI PF-06463922具有高于克唑替尼的ROS-1抑制活性和CNS穿透性，对克服克唑替尼耐药有效。

本研究纳入2例ROS-1阳性患者，其中1例ALK/ROS1同时阳性者克唑替尼疗效SD，PFS为6个月，OS已达13个月；另1例ROS-1阳性患者，克唑替尼疗效PR，PFS也为6个月，克唑替尼进展后继服克唑替尼+局部治疗，5个月后出现脑膜转移症状，开始应用三代ALK-TKI PF-06463922后第3日神志转清，症状好转，病情进展延迟，OS达14个月，支持PF-06463922可以克服ROS-1阳性NSCLC对克唑替尼的继发耐药。

关于克唑替尼的安全性：既往几个大型国际临床试验数据^[3, 5, 13]^表明，克唑替尼总的耐受良好，大多数不良反应为1级或2级，因不良反应退组的发生率较低。常见不良反应包括消化道反应（恶心、呕吐、腹泻、便秘、食欲减退）、视觉紊乱、转氨酶升高、粒细胞减少以及水肿、疲乏等，其中最常见的3级/4级毒性为转氨酶升高、粒细胞减少等。少见的不良反应有：心动过缓、Q-T间期延长、贫血等。罕见可能较严重的不良反应为致命性肝毒性（＜1%）、间质性肺病（1.6%），治疗中需监测肝功能、肺毒性以指导减量或停药。

本组研究的不良反应与国际研究大致相似，发生率由高到底依次为消化道反应、转氨酶升高、视觉异常、粒细胞减少、疲乏等，前4种不良反应除视觉异常外均包含3级/4级毒性，3级/4级毒性发生率与国际报道接近（如3级/4级转氨酶升高发生率7.5%，与PROFILE 1001和1005共计3级以上的转氨酶升高发生率7.4%^[3]^及亚裔患者约5%相似），患者因此减量和暂时停药，经对症治疗后均可恢复治疗，没有因此终止治疗者，即使1例应用二代ALK-TKI期间出现间质性肺炎这一严重毒性的病例，经停药20天和激素治疗后症状明显好转，未影响继续治疗，更换三代ALK-TKI后无复发。本组1例听觉异常既往研究未曾报道，因该患者存在CNS转移病灶，不能肯定与药物治疗相关。本组的视觉紊乱发生率43%，特异表现为视物模糊、夜间视物闪光、拖尾、复视、视力下降，均可耐受，部分患者在开始服药1个月-2个月内感觉明显，之后减轻。这与国际报道的情况类似。

综上所述，本研究证实了克唑替尼作为ALK/ROS-1抑制剂在ALK重排和ROS-1阳性NSCLC中患者一线及多线治疗的临床疗效，验证了该组患者临床及病理特征、近远期疗效及安全性数据与国际研究的结果相近。克唑替尼一、二线治疗效果更优，脑转移进展是克唑替尼治疗后进展的常见形式。孤立性脑转移进展患者可采用继续克唑替尼治疗联合脑局部放疗的策略，克唑替尼耐药患者可考虑接受二代/三代ALK抑制剂治疗。克唑替尼总体安全性良好，主要不良反应包括消化道反应、转氨酶升高、粒细胞减少和特征性视觉效应，治疗中需监测血象、肝、肺毒性以调整用药。ALK/ROS-1抑制剂是目前ALK、ROS-1阳性分子亚型肺癌的最佳治疗选择，是继EGFR-TKI后肺癌分子靶向治疗领域的又一个飞越性成就。
